# Evaluation of *N*-Alkyl-bis-*o*-aminobenzamide Receptors for the Determination and Separation of Metal Ions by Fluorescence, UV-Visible Spectrometry and Zeta Potential

**DOI:** 10.3390/molecules24091737

**Published:** 2019-05-04

**Authors:** Marisela Martinez-Quiroz, Xiomara E. Aguilar-Martinez, Mercedes T. Oropeza-Guzman, Ricardo Valdez, Eduardo A. Lopez-Maldonado

**Affiliations:** 1 CETYS Universidad, Centro de Innovación y Diseño, Escuela de Ingenieria Av. CETYS Universidad No. 4 Fracc. El Lago, Tijuana, B.C. CP 22210, México; 2 Tecnologico Nacional de México, Instituto Tecnológico de Tijuana, Blvd. Alberto Limón Padilla s/n, Mesa de Otay, Tijuana, B.C. CP 22500, México; xiomaraeam@gmail.com (X.E.A.-M.); oropeza1@yahoo.com (M.T.O.-G.); 3 Centro de Nanociencias y Nanotecnología CNyN-UNAM, Km 107 Carretera Tijuana-Ensenada, Ensenada, B.C. CP 22860, México; ricardovc1030@gmail.com; 4 Facultad de Ciencias Químicas e Ingeniería, Universidad Autónoma de Baja California, Tijuana, B.C. CP 22390, México

**Keywords:** zeta potential, aminobenzamides, metal titration, fluorescent probes

## Abstract

This paper presents the synthesis and evaluation of physicochemical behavior of a new series of *N*-alkyl-bis-*o*-aminobenzamides (BOABs) in aqueous solution. The study was targeted to the complexing capacity of five metal ions (Fe^2+^, Cu^2+^, Cd^2+^, Hg^2+^ and Pb^2+^) of environmental concern as the medullar principle of a liquid phase sensor for its application in the determination of these metal ions due to its versatility of use. Molecular fluorescence, UV-visible and Zeta potential were measured for five BOABs and the effect of alkyl groups with different central chain length (*n* = 3, 4, 6, 8 and 10) on physicochemical performance determined. The results have shown that these derivatives present higher sensibility and selectivity for Cu^2+^ even in the presence of the other metal ions. An additional application test was done adding a pectin (0.1 wt %) solution to the BOAB-Cu^+2^ complex to obtain a precipitate (flocs) as a potential selective separation process of Cu from aqueous solution. The solid was then lyophilized and analyzed by SEM-EDS, the images showed spheric forms containing Cu^+2^ with diameter of approximately of 8 μm and 30 wt %.

## 1. Introduction

The first fluorescent probe was reported in 1987 based on a sulfonamide compound [1]. Different sensors for cations of biological or environmental importance using fluorescent probes followed this work [2]. These sensors are typically composed of a spacer and a fluorescent compound, and there are several reported structures [3,4,5]. In recent years, the development of sensors has been expanded for its broad field of action. Recently, the design and development of selective and sensitive fluorescent molecules capable of detecting transition and metal ions have attracted considerable attention because of their use and subsequent impact on the environment and nature [6]. A large number of receptors has been study for the detection of metal ions. The basis of molecular recognition lies in the interaction of the metal-ligand, which is the weak binding of metal ions to one or more heteroatoms (nitrogen or oxygen) within the ligand and intramolecular energy transfer from the ligand to the metal ion [6,7,8].

It is known that bis-*o*-aminobenzamides has been a subject of study in the recognition of metal ions in the solid phase in previous cases and the synthesis on Merrifield’s resin and the addition of different ions obtained good results for Mg^2+^ [9]. Other studies show only the synthesis routes and characterization of this type of compound [10,11]. Additionally, aminobenzamide derivatives are used as pharmacological inhibitors on Parkinson’s disease [12], human cancer cells [13], and neurobehavioral and neurochemical anomalies [14]. The development of fluorescent sensors has received appreciable attention for detecting biologically important ions, either cations or anions [15].

There are no reports of the use of the bis-*o*-aminobenzamides derivatives on the determination and separation in solution of metal ions for environmental applications, only for biological applications. Hence the importance of the implementation of new bis-*o*-aminobenzamides compounds capable of being used in the detection and separation of metal ions in water treatment. It is expected that the use of these materials provide different properties as chemosensors and the recognition of the metal ions with advantages for the development of new materials with chelant-coagulant-flocculants properties, considering the effects and influences observed on diverse spectral changes due to the photophysical characteristics of these derivatives.

In this paper, the physicochemical behavior of a new series of *N*-alkyl-bis-*o*-aminobenzamides with different central chain lengths were studied in aqueous solution and their complexing capacity with different metal ions (Fe^2+^, Cu^2+^, Cd^2+^, Hg^2+^ and Pb^2+^) of environmental importance evaluated. The techniques of fluorescence, UV-Vis and zeta potential allowed to systematically complement the physicochemical evaluation of the BOABs and determine their strategic application.

## 2. Results and Discussion

### 2.1. Fluorescence and UV-Vis Properties of Compounds 1a–5a

**Figure 1:**
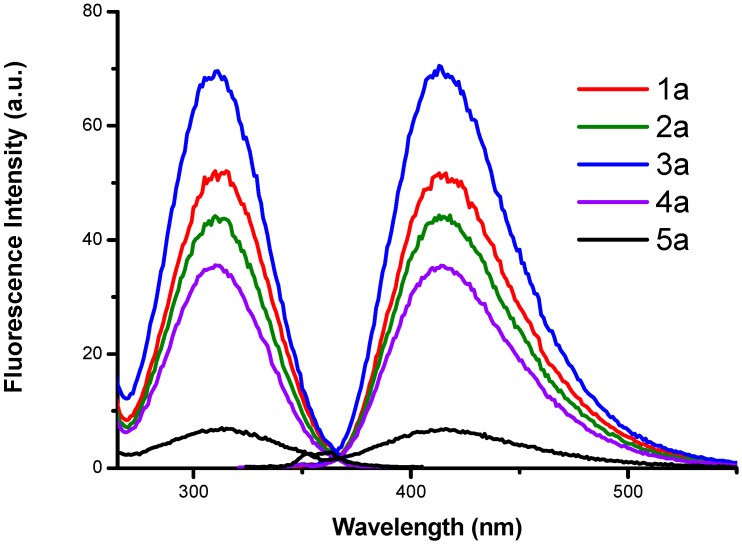
Fluorescence spectra of *N*-alkyl-bis-*o*-aminobenzamide **1a**–**5a** [1 × 10^−5^ M] in water at pH = 5.4.

The fluorescence spectra were obtained for derivatives **1a**–**5a** in order to evaluate how the central chain length influences the spectral characteristics of these compounds. The excitation and emission fluorescence spectra of the different derivatives in aqueous solution are presented in Figure 1.

The excitation and emission maximum wavelengths (λ_exc_ = 312 nm, λ_em_ = 413 nm) regardless of the alkyl chain length) all derivatives showed the same fluorescence peak position. The compound showing a higher fluorescence intensity is 3a which has a central alkyl chain length of six carbons, due to the fact the length of the chain increases the interaction of the molecule with the medium and the internal conversion velocity increases, there decreasing the fluorescence for the derivatives **4a** and **5a**, favoring the conformational effect of the more stable complexes, while in the other compounds it does not appear due to the mobility of the molecule [16,17,18]. Substituents have large effects on fluorescence that help to delocalize π electrons, such as –NH_2_ groups, that increase the fluorescence because they tend to increase the probability of transitions between the lowest singlet state and the base state. Molecular rigidity reduces the interactions of a molecule with its surrounding environment and, consequently, reduces the speed of deactivation by collisions (external conversion) in the series of compounds, those that are flatter, more rigid and more hindered, are the most fluorescent. The derivatives showed the following order **3a** > **1a** > **2a** > **4a** > **5a** according to characteristics and structural changes reflected by fluorescence intensity.

**Figure 2:**
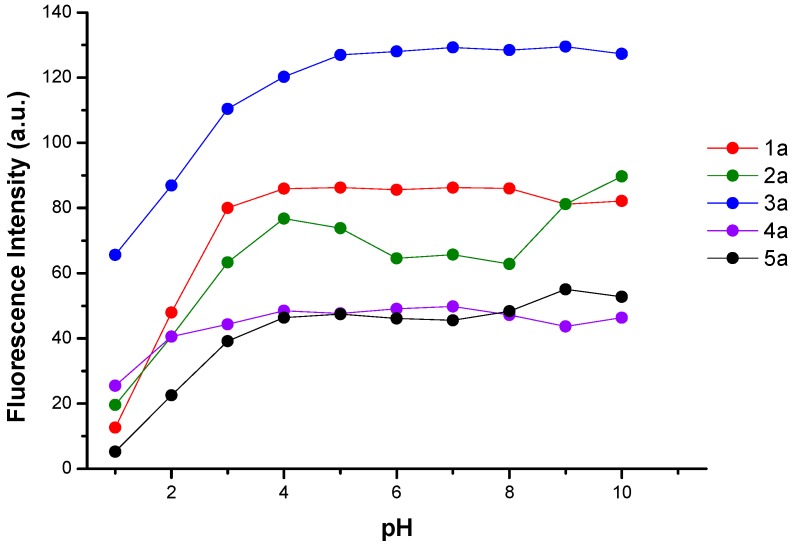
Effect of pH of *N*-alkyl-bis-*o*-aminobenzamide **1a**–**5a** at maximum emission [1 × 10^−5^ M].

The luminescence properties of *N*-alkyl-bis-*o*-aminobenzamide in solution showed similarity to reported studies in solid phase and the ability to delocalize charge over the carbonyl-benzyl-amine group [4]. However, in this study it is presented that these BOABs derivatives in aqueous solution have the same fluorescence response without being supported on the resin. This gives it an advantage for the application as a sensor in the detection of metal ions and as separation agents in wastewater treatment. As shown in Figure 2 the fluorescence of these derivatives is sensitive to pH changes. It can be observed that the fluorescence increases with increasing the pH, this behavior is attributed to the pka values of the derivatives, which at pH > pka the characteristic functional groups ionize and favor charge transfer (see Table 1). These derivatives have a negative surface charge at pH > IEP (isoelectric point) and positive charge at pH < IEP and different limit of ζ values. On the other hand, the pK_a_ values for derivatives **1a**–**5a** were calculated using Excel Worksheets for Spectrometry [19].

In Table 2, the photophysical parameter of the derivatives are shown. The quantum yield or the quantum efficiency of fluorescence is the ratio between the numbers of molecules that emit radiation with respect to the total number of molecules excited. Under certain conditions, for highly fluorescent molecules the quantum yield approaches unity, while in non-fluorescent molecules it is practically zero. The quantum yields (Φ*_F_*) in water at 1 × 10^−5^ M were determined following Equation (1):

(1) $$\Phi_{F}=\Phi_{R}\;X\frac{Int\;AR\;n^{2}}{{Int}_{R}A{n^{2}}_{R}}$$

where Φ*_F_* is the compound quantum yield, Int is the area under the emission peak, A is the absorbance at the excitation wavelength, and n is the refractive index of the sample. The subscript R denotes the respective values of the reference substance. The derivative **3a** is the one that had the highest quantum yield (Φ*_F_* = 0.0315), which enhances its functionality as a fluorescent sensor, unlike the derivative **4a** that had the lowest (Φ*_F_* = 0.0015).

**Figure 3:**
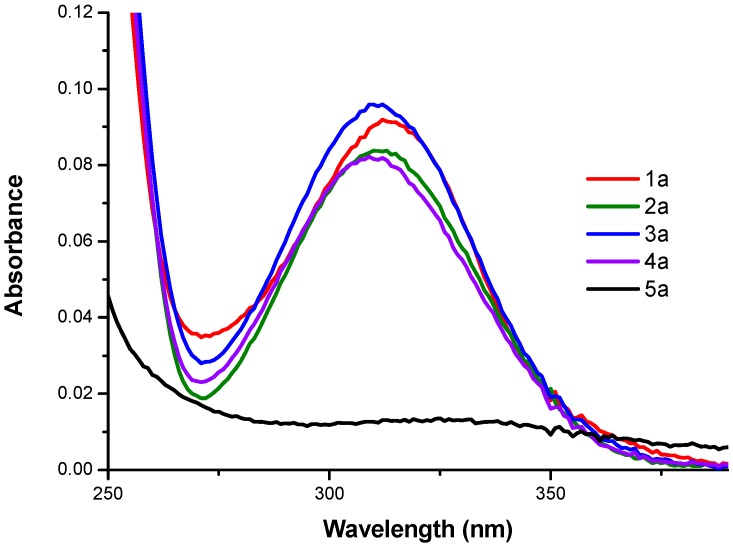
Absorbance spectra of *N*-alkyl-bis-*o*-aminobenzamides **1a**–**5a** [1 × 10^−5^ M] in water at pH = 5.4.

The UV-Vis spectra of the derivatives were obtained to complement and corroborate the photophysical properties determined by the fluorescence studies at the same pH value. In Figure 3 the absorbance spectra obtained are shown, where the derivatives show the order **3a** > **1a** > **2a** > **4a** > **5a** according to characteristics and structural changes by UV-Vis.

### 2.2. ζ = f (pH) Profiles of the Compounds 1a–5a

**Figure 4:**
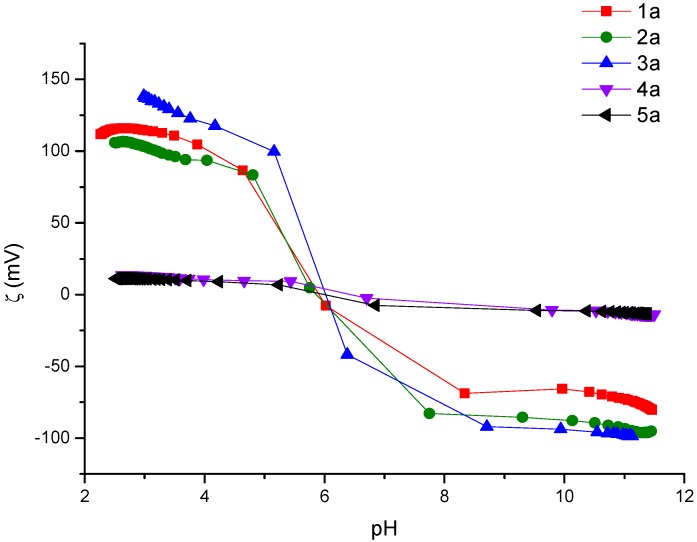
Profiles the ζ = f (pH) of BOAB compounds in water, concentration is 1 × 10^−5^ M.

The effect of pH on the zeta potential of compounds was studied in a pH range 2–12. Figure 4 shows two groups of zeta potential responses for the derivatives **1a**–**3a** and **4a**–**5a**. The first group have a positive charge and the IEP (isoelectric point) at pH~ 6, while the second group have a slightly positive charge and the IEP showed at pH~7. The change is observed in the pH value of the isoelectric point (IEP) depending of the long of the central chain. Table 1 shows IEPs for all derivative suggesting a clear influence of the central alkylic chain length. These derivatives have a negative surface charge at pH > IEP and positive charge at pH < IEP and different limit of **ζ** values. This characteristic presented by the derivatives can favor the formation of complexes, by favoring the interactions between metal ions.

### 2.3. Study of Metal Ions Recognition

**Figure 5:**
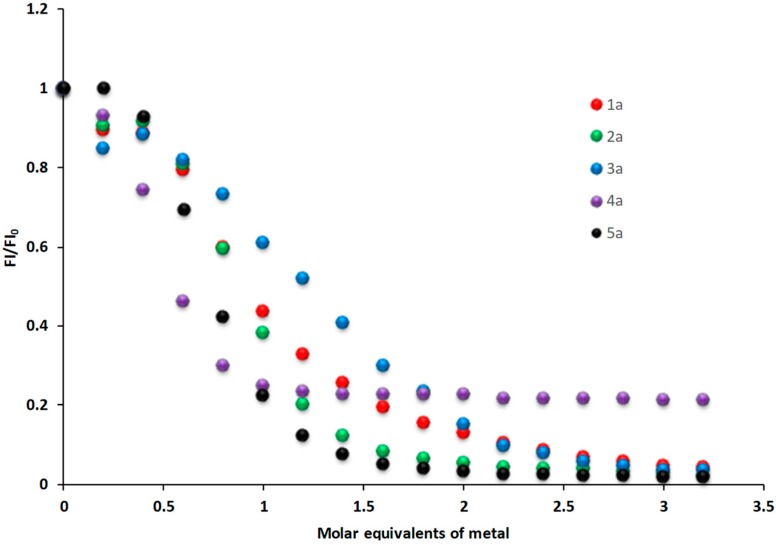
Relative fluorescence profiles obtained with Cu^2+^ and derivative **1a**–**5a** λ_ex_ = 312 nm, λ_em_ = 413 nm.

In order to evaluate the recognition capacity of the derivative **1a**–**5a** the corresponding relative fluorescence profiles were obtained in water with regard to its application as sensor and the fluorescence variation of the derivatives as a function of added metal ion is shown in Figure 5.

The metal ions were used Hg^2+^, Pb^2+^, Cd^2+^, Fe^2+^ and Cu^2+^. Derivatives **1a**–**5a** were titrated three times by successive increment of metal ions and the changes in the fluorescence were monitored. The addition of Hg^2+^, Pb^2+^, Cd^2+^and Fe^2+^ caused no variation in the fluorescence intensity. According the Pearson’s theory (Hard and Soft Acid Base theory, HSAB), functional groups of the BOABs have complementary affinities for metal ions: benzamide groups (O ligand atom) are considered as hard ligands with high affinity for hard ions (i.e, alkaline earth metals) while amine groups (N ligand atoms) are classified as intermediate ligand with higher affinity for intermediate ions (i.e., transition metal ions). The addition of Cu^2+^ ions causes a significant fluorescence quenching (72.3% average of FI initial) in all derivatives **1**–**5**, the stoichiometry of the metal ions coordination is 2:1 for **1a** and **3a**, 3:2 for **2a** and **5a**, 1:1 to derivative **4a**.

**Figure 6:**
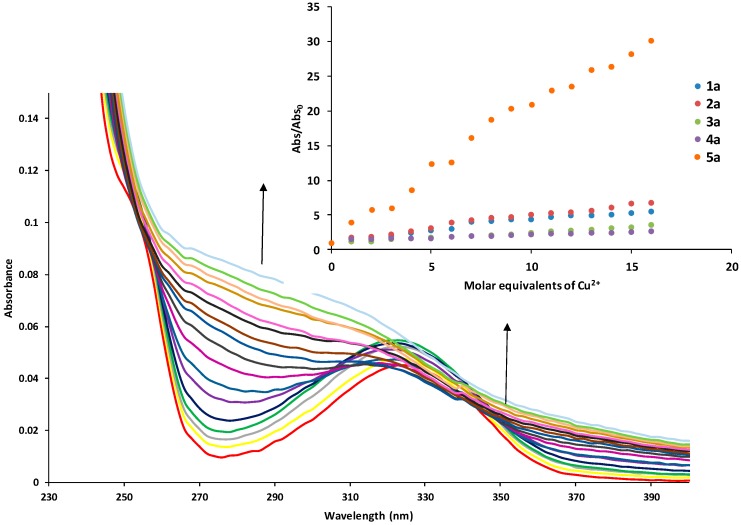
UV-Vis spectra obtained in the titration of decyl-derivative [1 × 10^−5^ M] and Cu^2+^ [1 × 10^−3^ M]. In set normalized absorbance profiles obtained by the titration of the derivative with Cu^2+^, λ = 316 nm.

UV-Vis spectra obtained in the titration of all derivatives [1 × 10^−5^ M] and Cu^2+^ [1 × 10^−3^ M] showed in all the situations the appearance of a new band, derivative **5a** was selected as an exemple because it shows a more evident change than the other derivatives. Figure 6 shows only UV-Vis spectra obtained in the titration of the decyl-derivative and Cu^2+^. The inset shows the UV-Vis spectra for derivative **1**–**5** obtained by titration with Cu^2+^. For the derivatives **1a**–**4a** there is a slight increase, and derivative **5a** showed significant changes in the absorbance due to interactions between the derivative and metal ions [20]. Other metal ions do not show interactions with the compounds. These results showed the evidence that derivative **3a** is indicated for analytical applications, while for implementation in water treatment it is derivative **5a**.

**Figure 7:**
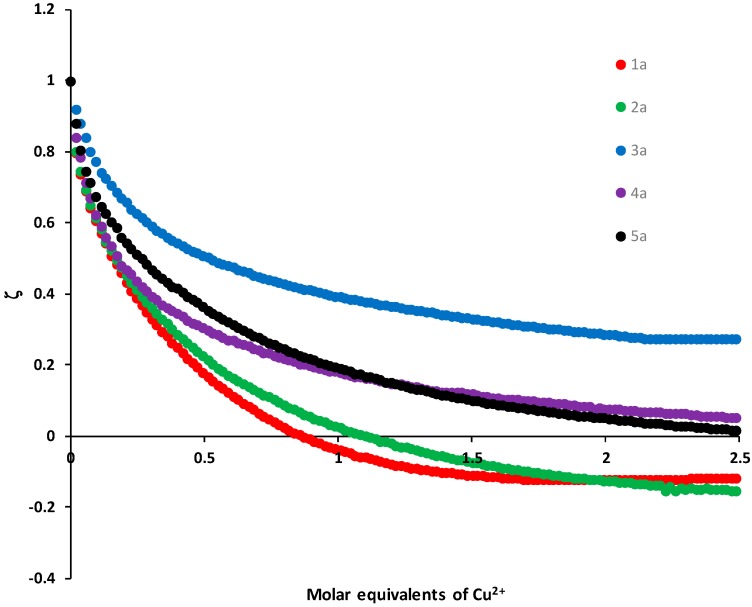
Normalized zeta potential profiles obtained by the titration of the derivative with Cu^2+^ [1 × 10^−3^ M].

The derivatives **1a**–**5a** in the presence of different metal ions of environmental importance were studied by zeta potential. These derivatives were titrated by successive additions of metal ion equivalents and the zeta potentials were measured. The addition of Cd^2+^, Fe^2+^, Hg^2+^ and Pb^2+^ did not cause a significant variation in the zeta potential. However, the addition of Cu^2+^ produced a significant change in the zeta potential for the derivatives **1a** and **2a**, followed by **4a** and **5a** (Figure 7).

The binding constants for derivatives **1a**–**5a** and Cu^2+^ were determined and are shown in Table 3, the association constant for the complexes calculated from the zeta potential data using Equation (2). These results show the stability of the complexes in aqueous media, the values obtained are in the range of 10^5^ to 10^9^.

Derivative **3a** has a value of k_f_ = 2.2 × 10^7^ intermediate, while compound **2a** showed the lowest value of k_f_ = 3.28 × 10^5^. This implies that the complexes are more stable Cu-**3a** and allows a better capacity (considering the value of k_f_) to sense Cu^2+^ and its possible application as selective agents of Cu separation in wastewater [21,22,23]. For the derivative **4a** the data obtained was not adjusted to perform the calculation:

(2) $$\zeta =\zeta_{H}+0.5\;\zeta_{\infty }\left\{{\left[H\right]}_{T}+{\left[G\right]}_{T}+\frac{1}{K}\;\sqrt{{\left({\left[H\right]}_{T}+{\left[G\right]}_{T}+\frac{1}{K}\right)}^{2}-4{\left[H\right]}_{T}\times {\left[G\right]}_{T}}\right\}$$

where:

ζ = Complex zeta potential
ζ_H_ = Free receptor zeta potential
ζ_∞_ = Zeta potential in the saturation point induced by complexation
[*G*]*_T_* = Receptor concentration
[*H*]*_T_* = Metal ion concentration
*k* = Association constant

### 2.4. Interference Study of the Copper Sensor

**Figure 8:**
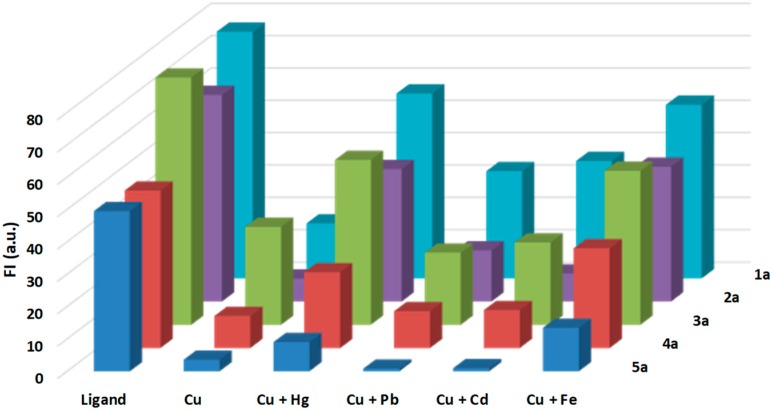
Fluorescence intensity profile of **1a**–**5a** [1 × 10^−5^ M] in water in the presence of selected metal ions [1 × 10^−3^ M].

The selectivity of the derivatives **1a**–**5a** toward Cu^2+^ ions over other competitive species was studied in the presence of various environmentally significant metal ions. All the measurements were conducted by using solutions of metal ions [1 × 10^−3^ M] in water. As illustrated in Figure 8 the results show an excellent selectivity of the proposed Cu^2+^ sensor in the presence of the mentioned metal ions.

In particular, derivatives **3a** and **5a** showed a better sensory capacity for Cu and for the separation in the presence of other metal ions of environmental interest. All the compounds titrate at the same concentration of metal ions [1 × 10^−3^ M] (1 × 10^−3^ M Cu^2+^ and 1 × 10^−3^ M for the other ion).

**Figure 9:**
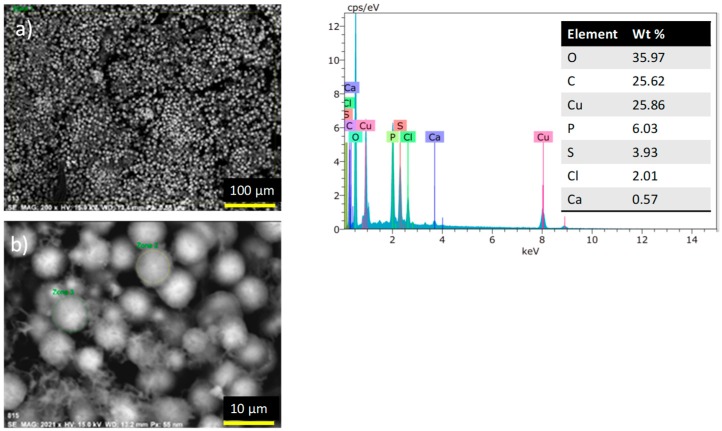
SEM-EDS image of complex **1a**-Cu, scale: (**a**) 100 μm, (**b**) 10 μm.

The results obtained by fluorescence, UV-Vis and zeta potential show a concordance and the possible application of the compounds in one of the stages of the water treatment according to the bifunctional effects shown. The detection of copper against that of other metals is evident, and in the SEM-EDS images obtained from the derivatives with metals the presence of microspheres of copper can be observed in the solid dry flocs (Figure 9). The SEM images showed evidence of the formation of supramolecular aggregates with *N*-alkyl-bis-*o*-aminobenzamide-copper-pectin and the possible application of these compounds in wastewater treatment.

## 3. Conclusions

The derivatives showed a high bifunctional performance for the detection and separation of metal ions of great environmental relevance. The measurements of zeta potential in combination with molecular fluorescence and UV-Vis allow us to evaluate their physicochemical behavior against the metal ions Fe^2+^, Cu^2+^, Hg^2+^, Pb^2+^ and Cd^2+^. Fluorescence is a technique that allows determining in a specific way with which metal ion the ligands have greater chemical affinity. However, the zeta potential measurements only allow to predict and approximate the electrostatic interactions between the BOABS and metal ions. The length of the alkyl chain (*n* = 3, 4, 6, 8 and 10) of the BOABs showed a significant effect on the fluorescence response, UV-Vis and zeta potential. Derivatives **3a** and **5a** are the compounds with the highest bifunctional performance. The monitoring of the complexation reaction of compound **3a**-Cu^2+^ by fluorescence allowed us to determine its stoichiometry and to enhance its application as a coagulating agent in the selective separation of Cu^2+^ by adding in a 1:1 ratio of a natural biopolyelectrolyte pectin. The studies have shown that these materials present higher sensibility and selectivity for Cu^2+^ (30%) even in the presence of other metal ions.

## 4. Materials and Methods

### 4.1. Materials

The materials were purchased from Sigma Aldrich (Mexico City, Mexico) in the highest available purity (>90%). NMR spectra were obtained in a Varian Mercury 200 MHz (Varian, Mexico). UV-Vis absorption spectra were obtained using a Cary 300 spectrophotometer (Agilent, Mexico). Fluorescence emission spectra were obtained using a Varian spectrofluorometer (Varian, Mexico). Deionized water (resistivity: 18.2 MΩ, MilliQ. Advantage A10, Merck, Mexico) was use for sample preparation in all experiments. All solvents were of spectroscopic or HPLC grade. Zeta Potential data was record on a Stabino Particle Charge Mapping device (Microtrac, Mexico). The measurements were done at room temperature in porcelain cuvettes. Sample solutions used to study the pH dependence of the potential zeta were prepared adjusting to the desired pH, with 0.1 M NaOH and 0.1 M HCl. The effect of metal ions upon the zeta potential was examined by adding a few microliters of stock solution (1 × 10^−3^ M) of the study metal ions to a known volume of the solution (3 mL). The addition was limited to 0.9 mL, so that dilution remained insignificant [24]. All the titrations and measurements they were three times. 

### 4.2. Synthesis

**Figure 10:**
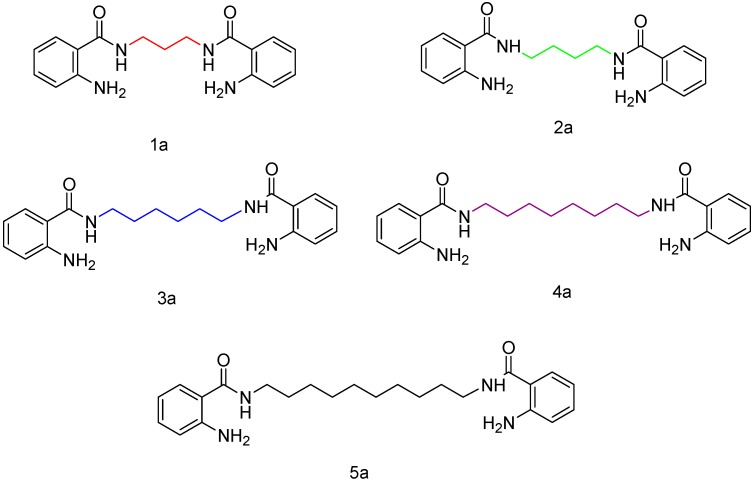
*N*-alkyl-bis-*o*-aminobenzamide derivatives **1a**–**5a.**

The derivatives **1a**–**5a** were synthesized and characterized according to the reported methodology (see Figure 10) [4]. 

*Propyl-bis-o-aminobenzamide* (**1a**). Isatoic anhydride (1.5 g) was dissolved in DMF (10 mL) and cooled to 0 °C. Then, 1,3-propylendiamine (0.34 g) was added dropwise under inert atmosphere conditions and the temperature was increased to 60 °C and stirred for 1 h. The reaction mixture was poured into hot water (50 mL), and the solid product was filtered and recrystallized from ethanol. A white solid was obtained (1.30 g, 90% yield). Mp. 170–172 °C. FT-IR: 3474, 3360, 3300, 3052, 1637, 1572, 1300, 1254, 1155 cm^−1^. ^1^H-NMR (DMSO-d_6_, 200 MHz): δ 8.17 (t, *J* = 5.4 Hz, 2H, NH), 7.57 (dd, *J* = 7.8, 1.3 Hz, 2H, H-6), 7.15 (ddd, *J* = 8.3, 8.2, 0.9 Hz, 2H, H-4), 6.68 (dd, *J* = 8.4, 0.9 Hz, 2H, H-3), 6.55 (ddd, *J* = 8.2, 7.8, 0.9, 2H, H-5), 6.33 (brs, 4H, NH_2_), 3.24 (td, *J* = 6.6, 5.4 Hz, 4H, CH_2_-α), 1.72 (q, *J* = 6.9 Hz, 2H, CH2-β). 

*Butyl-bis-o-aminobenzamide* (**2a**)**.** (1.40 g, 95%). Mp. 199–201 °C. FT-IR: 3482, 3375, 3294, 3053, 2936, 1624, 1584, 1320, 1266, 1158 cm^−1^. ^1^H-NMR (DMSO-*d*_6_, 200 MHz): *δ* 8.18 (t, *J =* 5.8, 2H, -N*H*), 7.44 (dd, *J =* 7.9, 1.2 Hz, 2H, H-6), 7.13 (ddd, *J =* 7.7, 7.2, 1.5 Hz, 2H, H-4), 6.68 (d, *J =* 7.5 Hz, 2H, H-3), 6.48 (dd, *J* = 7.8, 7.4 Hz, 2H, H-5), 6.37 (brs, 4H, N*H_2_*), 3.23 (brd, *J =* 5.6 4H, CH_2_-α), 1.54 (brs, 4H, CH_2_-β). 

*Hexyl-bis-o-aminobenzamide* (**3a**). (1.45 g, 90%), Mp. 163–165 °C. FT-IR: 3473, 3376, 3334, 3066, 2918, 1631, 1580,1542, 1317, 1260, 1156 cm^−1^. ^1^H-NMR (DMSO-*d*_6_, 200 MHz): *δ* 8.18 (brt, *J =* 5.4 Hz, 2H, N*H*), 7.46 (d, *J* = 7.9 Hz, 2H, H-6), 7.13 (ddd, *J =* 8.3, 7.1, 1.2 Hz, 2H, H-4), 6.65 (d, *J =* 8.3 Hz, 2H, H-3), 6.47 (dd, *J =* 7.9, 7.1 Hz, 2H, H-5), 6.39 (brs, 4H, N*H_2_*), 3.20 (td, *J =* 6.5, 6.1 Hz, 4H, CH_2_-α), 1.51 (brt, 4H, CH_2_-*β*), 1.36 (brs, 4H, CH_2_-*γ*). 

*Octyl-bis-o-aminobenzamide* (**4a**). (1.45 g, 83%), Mp. 175-177 °C. FT-IR: 3473, 3366, 3296, 3069, 2927, 1625, 1580, 1546, 1318, 1262, 1150 cm^−1^. ^1^H-NMR (DMSO-d_6_, 200 MHz): δ 8.2 (t, J) 5.5 Hz, 2H, NH), 7.45 (dd, *J* = 7.9, 1.5 Hz, 2H, H-6), 7.12 (ddd, *J* = 8.2, 7.7, 1.5 Hz, 2H, H-4), 6.67 (dd, *J* = 8.2, 1.1 Hz, 2H, H-3), 6.50 (ddd, *J* = 7.9, 7.7, 1.1 Hz, 2H, H-5), 6.39 (brs, 4H, -NH_2_), 3.19 (td, J) 6.5, 5.5, 4H, CH_2_-α), 1.50 (m, 4H, CH_2_-β), 1.30 (brs, 8H, H-CH_2_-γ,δ). 

*Decyl-bis-o-aminobenzamide* (**5a**). (1.45 g, 85%), Mp. 194–196 °C. FT-IR: 3475, 3366, 3296, 3069, 2927, 1625, 1580, 1546, 1318, 1262, 1150 cm^−1^. 

### 4.3. Procedure of Spectrometric Titrations

The effect of metal ions upon absorbance of derivatives **1a**–**5a** was examined by adding aliquots 6 µL (1 × 10^−3^ M) of metal solution to a known volume of compound solution 3 mL (1 × 10^−5^ M).

### 4.4. Fluorescence Measurements

Fluorescence measurements for derivatives **1a**–**5a** were carried out at room temperature in 1 cm quartz cuvettes in a Cary Eclipse Fluorescence Spectrophotometer. The samples (1 × 10^−3^ M) were dissolved in water solution. 

### 4.5. ζ = f (pH) Profiles of the Compounds ***1a***–***5a***

The potential zeta profiles of derivatives **1a**–**5a** were measures to evaluate the alkyl chain influence in the bifunctional performance, considering the surface charge characteristics of these derivatives. The measurements were done at room temperature in porcelain cuvettes. Influence of pH on the zeta potential behavior of **1a**–**5a** (1 × 10^−3^ M) derivatives was studied within a pH range of 2-11 with 0.1 M NaOH and 0.1M HCl.

### 4.6. Affinity Study of the Compounds ***1a***–***5a*** with Different Metal Ions

The chelating properties of the derivatives **1a**–**5a** in the presence of different metal ions (Cu^2+^, Pb^2+^, Fe^2+^, Hg^2+^ and Cd^2+^) were studied in aqueous solutions about their application as detector and new agents of separation for these environmental important metal ions. The derivatives were titrated by successive increment of equivalent number of these metal ions and the changes in the zeta potential values were measured.

## Figures and Tables

**Table 1:** Isoelectric point and pka’s of bis-*o*-aminobenzamides.

Compound	IEP	pka Fluorescence Values
**1a**	6	1.83
**2a**	6	2.69
**3a**	6	2.60
**4a**	7	3.32
**5a**	7	2.68

**Table 2:** Photophysical characteristics of **1a**–**5a** derivative.

Derivative/Parameter (nm)	λ_A_	λ_F_	λ_A_ − λ_F_	Φ_F_
**1a**	314	416	102	0.0026
**2a**	314	416	102	0.0023
**3a**	312	415	103	0.0315
**4a**	314	416	102	0.0015
**5a**	316	418	102	0.0049

**Table 3:** The binding constants (*k_f_*) of Cu^2+^ with the derivative **1a**–**5a**.

Compound	*k_f_*
**1a**	3.32 × 10^5^
**2a**	3.28 × 10^5^
**3a**	2.28 × 10^7^
**4a**	--
**5a**	1.10 × 10^9^
